# Risk factors for evisceration in gynecological oncology surgeries

**DOI:** 10.3906/sag-2004-333

**Published:** 2021-04-30

**Authors:** Fatih KILIÇ, Günsu KİMYON CÖMERT, Mehmet ÜNSAL, Çiğdem KILIÇ, Caner ÇAKIR, Dilek YÜKSEL, Alper KARALÖK, Osman TÜRKMEN, Taner TURAN

**Affiliations:** 1 Department of Gynecologic Oncology, Etlik Zübeyde Hanim Women’s Health Training and Research Hospital, Faculty of Medicine, University of Health Sciences, Ankara Turkey

**Keywords:** Evisceration, hypoalbuminemia, obesity, smoking, survival, wound dehiscence

## Abstract

**Background/aim:**

To investigate the risk factors for evisceration in a gynecological oncology population. The secondary aim was to evaluate the impact of evisceration on survival.

**Materials and methods:**

Inclusion criteria consisted of having had an elective surgery performed through a xiphoidopubic incision in our institution and having a gynecological malignancy based on pathology. A total of 198 patients were evaluated, 54 with evisceration and 144 without evisceration. Due to the widely varied prognosis of female genital cancers, the survival was analyzed on a homogenized group, including only 62 patients with primary advanced stage epithelial ovarian-tubal-peritoneal cancer.

**Results:**

The preoperative factors associated with evisceration in the univariate analysis were old age, high body mass index (BMI), hypertension, smoking, comorbidities, high American Society of Anesthesiologist (ASA) score (3 and 4), and low preoperative albumin level. The associated intraoperative factors were bleeding volume, receiving more than two units of erythrocyte suspension or fresh frozen plasma, and having had a major operation. The associated postoperative factors were the albumin transfusion and the antibiotic use in the early postoperative period. In the multivariate analysis, smoking, low levels of preoperative albumin, high BMIs, and high ASA scores (3 and 4) were independent prognostic factors for evisceration. Evisceration was not associated with recurrence and survival in the patients with primary advanced stage epithelial ovarian-tubal-peritoneal cancer.

**Conclusion:**

Smoking, preoperative hypoalbuminemia, obesity, and high ASA scores (3and 4) were the prognostic factors for evisceration. Short-term modifiable factors such as smoking cessation and improved nutritional status should be considered in elective gynecological oncology surgeries. Evisceration had no impact on survival and recurrence in the patients with primary advanced stage epithelial ovarian-tubal-peritoneal cancer.

## 1. Introduction

Laparotomy continues to be a vital procedure for emergency and elective surgeries despite the trend toward an increase in minimally invasive techniques to avoid the surgical site complications. Although laparotomy techniques have been improving, abdominal wound dehiscence is still a common postoperative surgical site complication with an incidence of up to 3.5% [1,2]. Furthermore, evisceration can cause serious morbidities in up to 45% of the cases [1,3–5].

Wound healing is a complicated process affected by several factors, such as patient characteristics, comorbidities, closure techniques, and materials [1,2,5]. The primary purpose of this study was to investigate the risk factors for evisceration in the patients undergoing gynecological oncology surgeries. The secondary aim was to evaluate the impact of evisceration on the survival of the patients with primary advanced stage epithelial ovarian-tubal-peritoneal cancer.

## 2. Materials and methods

### 2.1. Study design and patient selection

We reviewed the electronic data and files of the patients who underwent elective primary or recurrent surgeries due to the malignant gynecological diseases between January 1, 2005 and May 1, 2017, in the gynecological oncology clinic of our hospital. The institutional review board approved the study protocol (04.2017/07). Wound dehiscence was considered evisceration, which was defined as the loss of the integrity of the fascial closure in the abdominal area and did not include those with cutaneous separation. A total of 84 patients had evisceration. Those with incomplete data (n = 5), those with initial surgery performed in another center (n = 2), those with an incision that was not xiphoidopubic (n = 14), those without a gynecological malignancy in the final pathology report (n = 4), and those who underwent an emergency operation because of the surgical complications such as ileus, anastomotic leakage, and abscess (n = 5) were excluded. The study included those who had an elective surgery performed by vertical incision from the xiphoid process to the pubic bone in our institution and who had a gynecological malignancy such as cervical, endometrial, tubal, ovarian, or peritoneal carcinoma based on pathology. The final study group was formed with 54 patients with evisceration and 144 patients without evisceration who were operated on in the same period and had similar characteristics.

### 2.2. Data collection

The data related to the demographics, medical history, blood laboratory values, and the pre-, intra-, and postoperative clinical features were reviewed. The patients who smoked until the day before the operation were considered smokers. Chronic diseases (diabetes mellitus, hypertension, asthma, or other chronic diseases), smoking, and obesity (body mass index >30 kg/m2) were defined as comorbidities. Complete blood counts and biochemical analyses were obtained in the last 7 days before the operation and on the day after the operation. The laboratory parameters were presented as median values along with the reference levels used by our laboratory. American Society of Anesthesiologist (ASA) scores were evaluated by the anesthesiologists. For antibiotic prophylaxis, cefazolin was given to all patients 60 min before the incision. The additional doses of antibiotics were administered when the operation time exceeded 2 h or when there was an excessive blood loss during the surgery (>1500 mL). 

The type of the operation was categorized as follows: ‘simple’ for total abdominal hysterectomy with or without bilateral salpingo-oophorectomy; ‘complex’ for the surgeries with additional lymphadenectomy, appendectomy, or omental resection; and ‘major’ for the surgeries with the addition of any type of gastrointestinal resection, splenectomy, peritonectomy, diaphragm stripping, partial pancreatectomy, or liver resection. An antibiotic therapy in the first 24 h after the operation was defined as the initiation of antibiotic therapy in the early postoperative period. In the postoperative period, routine antibiotic administration is not used for the patients who undergo surgery in our clinic, except for intraoperative surgical prophylaxis. However, antibiotics can be administered in the early postoperative period (within the first 24 h after the operation), depending on the patient’s symptoms, infection markers, surgical procedure (gastrointestinal resection, anastomosis), and surgeon’s decision.

In all patients, abdominal fascial closures were performed by experienced gynecological oncology specialists. Synthetic nonabsorbable monofilament sutures were used for all fascial closures. The incisions were closed with continuous suture technique, placed at 2 cm lateral to the fascial edge with 1.5 cm bites.

### 2.3. Statistical analysis

Data were analyzed using SPSS version 20.0 for Windows (SPSS Inc., Chicago, United States). Categorical variables were compared using Pearson’s chi-square or Fisher’s exact test as appropriate. The factors with a possible effect on evisceration were evaluated using logistic regression analysis. Variables with a P-value <0.25 in the univariate analysis were used to develop a multivariate model for predicting the relationship between significant factors and evisceration. Variables that were correlated by more than 50% were excluded in a multivariate analysis using a Cox proportional hazards model. Hazard ratios (HR) and 95% confidence interval (CI) were also calculated for each independent variable.

### 2.4. Survival analysis

The time from the surgery to the recurrence or the last follow-up visit was defined as the disease-free survival (DFS). The time from the surgery to death, which resulted from the disease or the last follow-up visit, was defined as the disease-specific survival (DSS). The time from the surgery to death because of the disease, surgery, surgical complications, or the last follow-up visit was defined as overall survival (OS). Survival analysis was performed using the Kaplan–Meier method. Survival curves were compared using the log-rank test. In the survival analysis, the patients who had cancers other than ovary-tubal-peritoneal cancer, had early-stage (1 and 2) ovary-tubal-peritoneal cancer, received neoadjuvant therapy, or did not receive adjuvant chemotherapy, were excluded. Due to the widely varied prognosis of female genital cancers, the survival analysis group was homogenized to evaluate the effect of evisceration on the survival analysis more accurately. Therefore, the survival analysis was performed for the patients only with primary advanced stage (3 and 4) epithelial ovarian-tubal-peritoneal cancer. The level of statistical significance was set at P < 0.05.

## 3. Results

The median age of the entire cohort was 55.1 years (range: 27–78). The histopathological origins of the tumors were in the ovaries, fallopian tube, or peritoneum in 114 (57.6%) patients, uterus in 75 (37.9%) patients, and cervix in 9 (4.5%) patients. Twenty-six (13%) patients underwent surgery due to the recurrence of gynecological malignancies. Comorbity was present in 154 (77.8%) patients. Twenty-four (12%) patients were smokers. Eighty-four (42.4%) patients had a history of previous abdominal surgery. The median time from the most recent operation to the current operation was 90.7 months (range: 1–600 months). The most recent operation was within the previous 6 months for 30 out of 84 (35.8%) patients. Thirty-six (18.2%) patients had a history of chemotherapy or radiotherapy. The ASA score was 3 and 4 in 85 (43%) patients. The current operation was due to recurrent disease in 26 (13%) patients. The median operation time was 317.6 min (range: 105–660 min). Forty-one (20.7%) patients had ascites. The operation type was simple or complex in 100 (50.5%) patients and major in 98 (49.5%) patients. Antibiotic treatment was given to 68 (34.3%) patients in the early postoperative period. The incidence of evisceration was 3.2% of the 2106 patients who underwent laparotomy with vertical midline incision during the period covered by this study. Preoperative, intraoperative, and postoperative findings are shown in Tables 1–4.

**Table 1:** Features of the entire cohort.

	n	Median (range)
Preoperative parameters		
Body mass index (kg/m2)	198	30.7 (18.3–51.1)
The time from main operation to the next operation but one	84	24 (1–600)
Preoperative given erythrocyte suspension (unit)	8	2 (1–3)
Preoperative given fresh frozen plasma (unit)	1	2
The time when the erythrocyte suspension was performed preoperatively (day)	8	1 (1–3)
Hemoglobin level (g/dL)	198	12.4 (8.2–16.8)
Leukocyte count (/µL)	198	7575 (1450–21.800)
Neutrophil count (/µL)	198	5000 (960–19.910)
Platelet count (103/mm3)	198	309.5 (34–951)
Albumin level (g/dL)	118	4.2 (2.2–5.3)
Glucose level (mg/dL)	198	99.5 (69–274)
Intraoperative parameters		
The duration of operation (minute)	198	300 (105–660)
Ascites volume (mL)	41	2500 (200–10.500)
Bleeding volume (mL)	198	750 (100–5000)
Given colloid fluid quantity (mL)	198	5500 (1500–14.700)
Given crystalloid fluid quantity (mL)	198	4500 (1000–12.000)
Given colloid fluid quantity (mL)	193	1000 (500–3300)
Intraoperative given erythrocyte suspension (unit)	88	2 (1–6)
Intraoperative given fresh frozen plasma (unit)	63	2 (1–4)
Postoperative parameters		
Postoperative given erythrocyte suspension (unit)	61	2 (1–3)
Postoperative given fresh frozen plasma (unit)	12	2 (1–2)
The time when the erythrocyte suspension was performed postoperatively (day)	61	3 (1–30)
Hemoglobin level (postoperative 1st day) (g/dL)	198	10.65 (7–15.8)
Leukocyte count (postoperative 1st day) (/µL)	198	12600 (1330–33500)
Neutrophil count (postoperative 1tst day) (/µL)	198	10445 (1130–28000)
Platelet count (postoperative 1st day) (103/mm3)	198	266.5 (51–721)
Albumin (postoperative 1st day) (g/dL)	59	2.4 (1.2–3.2)

**Table 2:** Relation between preoperative parameters and evisceration in the entire cohort.

Parameters		Evisceration				OR	95% CI	P-value
		Present		Absent				
		n	%	n	%			
Age (year) 1	≤56	22	21	83	79	1 (ref)	1.048–3.737	0.034
	>56	32	34.4	61	65.6	1.979		
Body mass index (kg/m2) 1	≤30.7	20	19.8	81	80.2	1 (ref)	1.149–4.158	0.016
	>30.7	34	35.1	63	64.9	2.186		
Diabetes mellitus	Absent	40	25.6	116	74.4	1 (ref)	0.695–3.025	0.320
	Present	14	33.3	28	66.7	1.450		
Hypertension	Absent	22	19	94	81	1 (ref)	1.439–5.197	0.002
	Present	32	39	50	61	2.735		
Asthma	Absent	51	27.1	137	72.9	1 (ref)	0.287–4.623	0.842
	Present	3	30	7	70	1.151		
Smoking	Absent	41	23.6	133	76.4	1 (ref)	1.597–9.205	0.002
	Present	13	54.2	11	45.8	3.834		
Comorbidity	Absent	6	13.6	38	86.4	1 (ref)	1.136–7.240	0.021
	Present	48	31.2	106	68.8	2.868		
History of the abdominal operation	Absent	32	28.1	82	71.9	1 (ref)	0.482–1.716	0.769
	Present	22	26.2	62	73.8	0.909		
The time of the most recent previous operation 1, 2	>24	11	28.2	28	71.8	1 (ref)	0.311–2.181	0.696
	≤24	11	24.4	34	75.6	0.824		
The time of the most recent previous operation (month) 2	>6	14	25.9	40	74.1	1 (ref)	0.377–2.860	0.941
	≤6	8	26.7	22	73.3	1.039		
Presence of surgery within preoperative 6 months 3	No	46	27.4	122	72.6	1 (ref)	0.401–2.319	0.936
	Yes	8	26.7	22	73.3	0.964		
History of chemotherapy or radiotherapy	No	45	27.8	117	72.2	1 (ref)	0.378–1.986	0.735
	Yes	9	25	27	75	0.867		
Surgery type	Primary	49	28.5	123	71.5	1 (ref)	0.213–1.674	0.323
	Recurrent	5	19.2	21	80.8	0.598		
ASA score	1 and 2	20	17.7	93	82.3	1 (ref)	1.619–5.934	<0.0001
	3 and 4	34	40	51	60	3.100		
History of preoperative transfusion of erythrocyte suspension	No	51	26.8	139	73.2	1 (ref)	0.377–7.090	0.507
	Yes	3	37.5	5	62.5	1.635		
Amount of the erythrocyte suspension (unit) 1	≤2	3	42.9	4	57.1	1 (ref)	NC	NC
	>2	0	0	1	100	NC		
Amount of the fresh frozen plasma (unit) 1	≤2	1	100	0	0	1 (ref)	NC	NC
	>2	0	0	0	0	NC		
Hemoglobin level (g/dL) 1	>12.4	24	24.5	74	75.5	1 (ref)	0.705–2.477	0.384
	≤12.4	30	30	70	70	1.321		
Hemoglobin level (g/dL) 4	>12	27	24.1	85	75.9	1 (ref)	0.769–2.701	0.254
	≤12	27	31.4	59	68.6	1.441		
Leukocyte count (/µL) 1	≤7575	24	24.2	75	75.8	1 (ref)	0.725–2.547	0.338
	>7575	30	30.3	69	69.7	1.359		
Leukocyte count (/µL) 4	≤10.000	43	26.4	120	73.6	1 (ref)	0.578–2.830	0.543
	>10.000	11	31.4	24	68.6	1.279		
Neutrophil count (/µL) 1	≤5000	27	27.3	72	72.7	1 (ref)	0.535–1.869	1.000
	>5000	27	27.3	72	72.7	1.000		
Neutrophil count (/µL) 4	≤8000	47	26.3	132	73.7	1 (ref)	0.609–4.408	0.325
	>8000	7	36.8	12	63.2	1.638		
Platelet count (103/mm3) 1	≤309.5	27	27.3	72	72.7	1 (ref)	0.535–1.869	1.000
	>309.5	27	27.3	72	72.7	1.000		
Albumin level (g/dL) 1	>4.2	6	10.9	49	89.1	1 (ref)	0.669–5.521	0.220
	≤4.2	12	19	51	81	1.922		
Albumin level (g/dL) 4	>3.5	11	10.7	92	89.3	1 (ref)	2.223–24.094	<0.0001
	≤3.5	7	46.7	8	53.3	7.318		
Glucose (mg/dL) 1	≤99.5	26	26.3	73	73.7	1 (ref)	0.592–2.070	0.750
	>99.5	28	28.3	71	71.7	1.107		

1: Median value, 2: among the 84 patients with history of abdominal operation, 3: Among the entire cohort (n: 198), 4: Reference level of our institution.

**Table 3:** Relation between intraoperative parameters and evisceration in the entire cohort.

Parameters		Evisceration				OR	95% CI	P-value
		Present(n: 54)		Absent(n: 144)				
		n	%	n	%			
The duration of theoperation (minutes)1	≤300	33	30.3	76	69.7	1 (ref)	0.376–1.345	0.294
	>300	21	23.6	68	76.4	0.711		
Ascites	No	38	24.2	119	75.8	1 (ref)	0.970–4.142	0.058
	Yes	16	39	25	61	2.004		
The amount of the ascites (mL) 1	≤2500	11	44	14	56	1 (ref)	0.155–2.165	0.414
	>2500	5	31.2	11	68.8	0.579		
Groups of the operation	Simple and Complex	20	20	80	80	1 (ref)	1.117–4.041	0.020
	Major	34	34.7	64	65.3	2.125		
Hysterectomy	Absent	10	27	27	73	1 (ref)	0.454–2.269	0.970
	Present	44	27.3	117	72.7	1.015		
Type of hysterectomy	Type 1	17	22.1	60	77.9	1 (ref)	0.825–3.390	0.152
	Type 2 and 3	27	32.1	57	67.9	1.672		
Oophorectomy(unilateral or bilateral)	Absent	9	25.7	26	74.3	1 (ref)	0.479–2.532	0.820
	Present	45	27.6	118	72.4	1.102		
Lymphadenectomy	Absent	7	25.9	20	74.1	1 (ref)	0.430–2.728	0.866
	Present	47	27.5	124	72.5	1.083		
Omentectomy	Absent	8	21.6	29	78.4	1 (ref)	0.617–3.407	0.392
	Present	46	28.6	115	71.4	1.450		
Appendectomy	Absent	32	22.7	109	77.3	1 (ref)	1.103–4.155	0.023
	Present	22	38.6	35	61.4	2.141		
Peritonectomy	Absent	49	28.5	123	71.5	1 (ref)	0.213–1.674	0.323
	Present	5	19.2	21	80.8	0.598		
Colon resection	Absent	35	23.2	116	76.8	1 (ref)	1.123–4.504	0.020
	Present	19	40.4	28	59.6	2.249		
Intestinal resection	Absent	52	27.5	137	72.5	1 (ref)	0.151–3.742	0.728
	Present	2	22.2	7	77.8	0.753		
Gastrointestinal system resection2	Absent	33	22.4	114	77.6	1 (ref)	1.226–4.769	0.010
	Present	21	41.2	30	58.8	2.418		
Diaphragm stripping	Absent	36	23.7	116	76.3	1 (ref)	1.028–4.173	0.039
	Present	18	39.1	28	60.9	2.071		
Splenectomy	Absent	43	24.9	130	75.1	1 (ref)	1.003–5.623	0.045
	Present	11	44	14	56	2.375		
Cholecystectomy	Absent	51	28.3	129	71.7	1 (ref)	0.140–1.822	0.289
	Present	3	16.7	15	83.3	0.506		
Hepatic resection	Absent	51	27.1	137	72.9	1 (ref)	0.287–4.623	0.842
	Present	3	30	7	70	1.151		
Amount of thebleeding (mL)1	≤750	20	20.2	79	79.8	1 (ref)	1.087–3.928	0.025
	>750	34	34.3	65	65.7	2.066		
Amount of the total fluid applied (mL) 1	≤5500	31	29	76	71	1 (ref)	0.441–1.558	0.560
	>5500	23	25.3	68	74.7	0.829		
Amount of the crystalloid fluid applied (mL) 1	≤4500	35	28.9	86	71.1	1 (ref)	0.420–1.542	0.513
	>4500	19	24.7	58	75.3	0.805		
Amount of the colloid fluid applied(mL)1	≤1000	33	23.9	105	76.1	1 (ref)	0.647–2.632	0.456
	>1000	16	29.1	39	70.9	1.305		
History of intraoperative transfusion of erythrocyte suspension	Absent	28	25.5	82	74.5	1 (ref)	0.656–2.300	0.521
	Present	26	29.5	62	70.5	1.228		
Amount of the erythrocyte suspension (unit) 1,3	≤2	10	17.9	46	82.1	1 (ref)	1.738–12.178	0.001
	>2	16	50	16	50	4.600		
Amount of the fresh frozen plasma(unit) 1,4	≤2	7	15.9	37	84.1	1 (ref)	1.138–12987	0.025
	>2	8	42.1	11	57.9	3.844		

1: Median value,2: including resection of stomach and/or intestines and/or colon, 3: among the 88 patients with history of the intraoperative transfusion of erythrocyte suspension, 4: among the 63 patients with history of the intraoperative transfusion of fresh frozen plasma.

**Table 4:** Relation between postoperative parameters and evisceration in the entire cohort.

Parameters		Evisceration				OR	95% CI	P-value
		Present		Absent				
		n	%	n	%			
History of postoperative transfusion of erythrocyte suspension1	Absent	37	26.1	105	73.9	1 (ref)	0.626–2.446	0.541
	Present	17	30.4	39	69.6	1.237		
Amount of the erythrocyte suspension (unit) 2	≤2	16	29.1	39	70.9	1 (ref)	NC	NC
	>2	1	100	0	0	NC		
Amount of the fresh frozenplasma (unit) 2	≤2	2	16.7	10	83.3	1 (ref)	NC	NC
	>2	0	0	0	0	NC		
The time of the applying the erythrocyte suspension (days) 2	≤3	12	36.4	21	63.6	1 (ref)	0.144–1.644	0.242
	>3	5	21.7	18	78.3	0.486		
Albumin transfusion	Absent	26	19.3	109	80.7	1 (ref)	1.741–6.461	<0.0001
	Present	28	44.4	35	55.6	3.354		
Hemoglobin, postoperative1st day (g/dL) 2	>10.65	27	27.3	72	72.7	1 (ref)	0.535–1.869	1.000
	≤10.65	27	27.3	72	72.7	1.000		
Hemoglobin, postoperative1st day (g/dL) 3	>12	12	24.5	37	75.5	1 (ref)	0.576–2.543	0.614
	≤12	42	28.2	107	71.8	1.210		
Leukocyte count, postoperative1st day (/µL) 2	≤12.600	26	26.3	73	73.7	1 (ref)	0.592–2.070	0.750
	>12.600	28	28.3	71	71.7	1.107		
Leukocyte count, postoperative1st day (/µL) 3	≤10.000	14	29.8	33	70.2	1 (ref)	0.413–1.749	0.658
	>10.000	40	26.5	111	73.5	0.849		
Neutrophil count, postoperative1st day (/µL) 2	≤10.445	29	29.3	70	70.7	1 (ref)	0.436–1.526	0.523
	>10.445	25	25.3	74	74.7	0.815		
Neutrophil count, postoperative1st day (/µL) 3	≤8000	13	33.3	26	66.7	1 (ref)	0.327–1.478	0.343
	>8000	41	25.8	118	74.2	0.695		
Platelet count, postoperative1st day (103/mm3) 2	≤266.5	28	28.3	71	71.7	1 (ref)	0.483–1.689	0.750
	>266.5	26	26.3	73	73.7	0.903		
Albumin level, postoperative1st day (g/dL) 2	>2.4	6	21.4	22	78.6	1 (ref)	0.838–8.368	0.092
	≤2.4	13	41.9	18	58.1	2.648		
The history of the early periodantibiotic usage	Absent	22	16.9	108	83.1	1 (ref)	2.253–8.451	<0.0001
	Present	32	47.1	36	52.9	4.364		

1: The erythrocyte suspension performed in the period between postoperative 1st day and occurrence of evisceration, 2: median value, 3: reference value of laboratory in our institution. NC: not calculated, OR: odds ratio, CI: confidence interval.

### 3.1. Preoperative factors

The median time from the current operation to the emergence of evisceration was 11 days (range: 1–44 days). The evisceration was associated with the following preoperative factors: old age, high body mass index (BMI), hypertension, smoking, any comorbidity, high ASA scores (ASA 3 and 4), and low preoperative albumin level. The odds ratios (OR) for evisceration were 1.98 (CI: 1.048–3.737; P = 0.034) for older age, 2.19 (CI: 1.149–4.158; P = 0.016) for high BMI, 2.74 (CI: 1.439–5.197; P = 0.002) for hypertension, 3.83 (CI: 1.597–9.205; P = 0.002) for smoking, 2.87 (CI: 1.136–7.240; P = 0.021) for the presence of any comorbidity, 3.1 (CI: 1.619–5.934; P < 0.0001) for high ASA scores, and 7.32 (CI: 2.223–24.094; P < 0.0001) for the presence of low albumin level at the preoperative period (Table 2). 

### 3.2. Intraoperative factors

The intraoperative parameters related to the evisceration were bleeding volume, receiving more than two units of erythrocyte suspension (ES) or fresh frozen plasma (FFP), and having had a major surgery (Table 3). The evisceration rate was 20.2% in the patients with intraoperative bleeding ˂750 mL while 34.3% in those with ˃750 mL bleeding volume (OR: 2.07, CI: 1.087–3.928; P = 0.025). The evisceration was not related to receiving ES during the operation. However, receiving more than two units of ES or FFP was related to an OR of 4.6 (CI: 1.738–12.178; P = 0.001) or 3.84 (CI: 1.138–12.987; P = 0.025) for evisceration, respectively. The evisceration rate was 20% for the patients with a simple or complex surgery compared to 34.7% for those with a major surgery (OR: 2.13, CI: 1.117–4.041; P = 0.020). Moreover, gastrointestinal system resection, colon resection alone, splenectomy, appendectomy, and diaphragm stripping were significantly associated with evisceration (Table 3).

### 3.3. Postoperative factors

In the univariate analysis, albumin transfusion and antibiotic usage in the early postoperative period were associated with a higher rate of evisceration (Table 4). The rate of evisceration was 44.4% in the patients who received postoperative albumin transfusion and 19.3% in those who did not (OR: 3.35, CI: 1.741–6.461; P < 0.0001). Antibiotic usage in the early postoperative period was also associated with evisceration (OR: 4.36; CI: 2.253–8.451; P < 0.0001). In the Cox proportional hazards model, smoking, a low level of preoperative albumin, high BMI, and high ASA scores (3 and 4) were independent prognostic factors for evisceration (Table 5).

**Table 5:** Multivariate analysis of parameters.

Parameters	Hazard Ratio	95% CI	P-value
Age (>56 vs. ≤56 years) 1	1.838	0.369–9.152	0.458
Smoking (present vs. absent)	22.129	2.494–196.329	0.005
Preoperative albumin level (≤3.5 g/dL vs. >3.5 g/dL) 2	11.798	1.938–71.829	0.007
Body mass index (>30.7 kg/m2 vs. ≤30.7 kg/m2) 1	6.062	1.301–28.260	0.022
Presence of hypertension (present vs. absent)	2.386	0.553–10.301	0.244
ASA score (3 and 4 vs. 1 and 2)	5.120	1.184–22.142	0.029
Presence ascites (positive vs. negative)	1.833	0.258–13.043	0.545
Groups of the operation (major vs. simple and complex)	3.843	0.866–17.056	0.077

1: Median value, 2: reference level of laboratory in our institution. ASA: American Society of Anesthesiologists, CI: confidence interval.

### 3.4. Evisceration and survival

The survival was analyzed for 62 patients with primary advanced stage (3 and 4) epithelial ovarian-tubal-peritoneal cancer. Evisceration occurred in 24 patients. The median follow-up time was 21 months (range: 1–108 months). Twenty-four (38.7%) patients had recurrence; 20 (32.3%) patients died. Two- and five-year survival rates were 55% and 25% for DFS, 95% and 62% for DSS, and 85% and 55% for OS, respectively.

The mean time from surgery to adjuvant chemotherapy was 28.6 ± 14.1 days (range: 10–65 days) for the patients with evisceration and 17.4 ± 5.7 days (range: 9–34 days) for those without (P < 0.0001). However, evisceration was not associated with recurrence and survival. The DFS was related to the stage of cancer only. The DSS was related to the type of tumor, the stage of cancer, and the recurrence time. The OS was related to the recurrence time alone (Table 6).

**Table 6:** The association between factors and survival, and recurrence among the 62 patients with primary advanced stage (3 and 4) epithelial ovarian-tubal-peritoneal cancer.

Parameters		n	2-year DFS(%)	P value	2-year CCS(%)	P-value	2 year OS (%)	P-value
Age (years) 1	≤58	31	53	0.995	92	0.579	86	0.192
	>58	31	58		75		84	
Histologic type of cancer	Serous	50	54	0.960	96	0.023	86	0.145
	Non-serous	12	60		88		78	
Stage	3	49	62	0.026	96	0.040	89	0.133
	4	13	31		77		71	
Evisceration	Not occurred	38	59	0.761	92	0.986	87	0.321
	Occured	24	50		91		81	
The time givenchemotherapy (days) 2	≤18	28	60	0.902	92	0.762	96	0.947
	>18	27	55		95		87	
The time givenchemotherapy (days)2	≤30	48	58	0.981	97	0.941	94	0.256
	>30	7	50		75		54	
Recurrence time(months)	≤18	12	-	-	82	0.010	82	0.010
	>18	12	-		88		88	
Recurrence time(months)	≤12	6	-	-	83	0.010	83	0.010
	>12	18	-		94		94	

1: Median value among the 62 patients, 2: Median value among the 55 patients. DFS: disease-free survival, OS: overall survival.

## 4. Discussion

The major finding of our study was that smoking, preoperative hypoalbuminemia, obesity, and high ASA scores were the independent prognostic factors associated with evisceration. Although the presence of evisceration caused a delay in initiation of the chemotherapy, it had no impact on survival. This is one of the few studies to evaluate risk factors for evisceration, as well as any association between evisceration and survival, in gynecological-oncologic patients.

Wound healing is a complex phenomenon that includes cellular, inflammatory, and proliferative processes [6]. A defect in this healing cascade may cause abdominal wound dehiscence. Various intrinsic and extrinsic factors can have an impact on dehiscence. However, these factors are still not clear. Studies have reported a diverse set of risk factors for wound dehiscence, including old age [3,4,7], male sex [3,7], emergency surgery [3,4,8], malignancy [4,6], wound infection [6,8], hypoproteinemia [3,4,6–9], obesity [4,7], intraabdominal sepsis [4], hemodynamic instability [4], ascites [4], anemia [7], long operation time [8], and high ASA scores [8]. 

The toxins in tobacco can delay and interrupt the wound healing process [10–12]. The underlying mechanisms are related to tissue hypoxia and reduced tissue perfusion since these toxins decrease the immune function and induce vasoconstriction, thrombogenesis, atherosclerosis, and a reduction in the oxygen-carrying capacity of the blood [12]. Therefore, the rate of the composite of wound-related complications including both wound dehiscence and surgical site infection is higher in smokers [10,11]. Goltsman et al. found that smoking was an independent risk factor for wound dehiscence and the risk of wound dehiscence significantly increased in smoker groups [10]. Dahl et al. reported that although surgical site infection was higher in smokers, there were no statistically significant differences for wound dehiscence between smokers and nonsmokers [11]. Similarly to Goltsman et al., our report showed that smoking was an independent risk factor for wound dehiscence.

Nutrition has a vital role in tissue repair due to the anabolic nature of healing [13]. Proteins are among the essential elements in nutrition as well as in wound healing [13]. Hypoalbuminemia is a significant indicator of malnutrition in the patients with gynecological cancers [14,15]. Although some studies have shown no association between wound dehiscence and hypoalbuminemia [1,16], several studies have shown hypoalbuminemia to be an independent prognostic factor for wound dehiscence [4,17–20]. Besides being a prognostic for morbidity, preoperative hypoalbuminemia is an adverse prognostic factor for survival in the patients with ovarian cancer [17,21]. Kenig et al. found no association between obesity (>30 kg/m2) and wound dehiscence [1]. Walming et al. and Nugent et al. determined that increasing BMI was associated with higher rates of evisceration [2,18]. Nugent et al. found that obesity, hypoalbuminemia, prior surgery, and pulmonary disease were major risk factors on a nomogram for wound dehiscence in a gynecological oncologic cohort [18]. The present study showed that preoperative hypoalbuminemia was a negative prognostic factor for evisceration. However, no association was found between hypoalbuminemia and survival in the present study. Our study supported both obesity and hypoalbuminemia as independent risk factors for evisceration. 

A high ASA score reflects several risk factors, such as old age, cardiovascular diseases, pulmonary diseases, obesity, and other chronic conditions. Additionally, a high ASA score may imply hypoxia, a suppressed immune system, and malnutrition. Nugent et al. and Novetsky et al. found increased incidences of wound dehiscence for the patients with ASA scores of 3 and above [18,22]. The present study supports the relationship between high ASA scores and increased wound dehiscence.

Wound dehiscence has long-term outcomes. The rate of incisional hernia was extremely high in the patients with a history of wound dehiscence [5]. The long-term quality of life scores indicated that physical and mental health were affected negatively in the patients with wound dehiscence [5,23]. Furthermore, besides high morbidity, wound dehiscence was associated with a high mortality rate ranging from 10% to 35% [1,5,7]. In the present study, no relationship was found between evisceration and survival or recurrence, although evisceration caused a delay in the initiation of chemotherapy

A significant limitation of the study was the retrospective design. Another possible limitation is that the data were obtained from a single center, although this might have minimized the impact of technical and physician-related factors. Other limitations were the inclusion of the recurrent patients, the diverse types of cancers (ovarian, cervix, uterine), and the patients with a history of abdominal operation or ascites, as they may impair wound healing. A major strength of the study was the exclusion of emergency operations. Additionally, the inclusion of patients with a single incision type (xiphoidopubic incision) and the large sample size that was sufficient for evaluating several parameters were among the other strengths of the study.

In conclusion, smoking, preoperative hypoalbuminemia, obesity, and high ASA scores were independent prognostic factors for evisceration. The minimization of these modifiable factors can decrease the risk of evisceration in elective gynecological surgeries. Furthermore, precautions such as the cessation of smoking and the rehabilitation of nutritional status should be considered, in the short term, for better outcome in these surgeries. Evisceration had no impact on survival and recurrence in the patients who underwent gynecological oncology surgeries.

## Informed consent

All participants were informed in the format requested by the relevant authorities and/or boards. The institutional review board approved the study protocol (04.2017/07).
